# Bilateral mydriasis as first manifestation of Hodgkin’s lymphoma: a case report

**DOI:** 10.1186/s12883-022-02941-6

**Published:** 2022-12-12

**Authors:** Enayatullah Baki, Klemens Scheidhauer, Friederike Schmidt-Graf

**Affiliations:** 1 Department of Neurology, Klinikum rechts der Isar, Technical University, Ismaningerstrasse 22, 81675 Munich, Germany; 2 Department of Nuclear Medicine, Klinikum rechts der Isar, Technical University, Ismaningerstrasse 22, 81675 Munich, Germany

**Keywords:** Mydriasis, Hodgkin’s lymphoma, Oculosympathetic spasm, Pericarotid sympathetic nerves

## Abstract

**Background:**

Bilateral mydriasis is usually associated with severe brain stem damage or drug-induced sympathomimetic stimulation.

Herein we report it as a unique neurologic complication of Hodgkin’s lymphoma.

**Case presentation:**

A 23-year-old woman presented at our emergency department with dilated pupils unresponsive to light stimuli. MRI and CT scans showed bilaterally enlarged lymph nodes in the mediastinum and supraclavicular compressing the carotid artery on both sides. The histologic examination of lymph node biopsy specimens confirmed the diagnosis of Hodgkin’s lymphoma.

**Conclusion:**

Pathologies around the carotid artery causing oculosympathetic spasm should be considered among the possible causes of a mydriasis, especially when other common causes like brain stem impairment are excluded.

## Background

Dilated and unreactive pupils are caused by parasympathetic paralysis or sympathetic stimulation. Reasons for a parasympathetic paralysis are often anticholinergic drugs like scopolamine or atropine [1] or oculomotor nerve palsy due to brain stem encephalitis, injury, ischemia or hemorrhage or local damage of the ciliary nerves [2].

Sympathetic stimulation can be a result of sympathomimetic drugs like cocaine or amphetamine [3].

Mydriasis as a result of impairment of the cervical sympathetic nerve chain is described in only very rare cases [4, 5] and has never been reported before as a neurologic complication of Hodgkin’s lymphoma.

Here, we present the case of a young woman with bilateral mydriasis as the first clinical manifestation of Hodgkin’s lymphoma and enlarged pericarotid lymph nodes causing oculosympathetic spasm.

## Case presentation

**Fig. 1:**
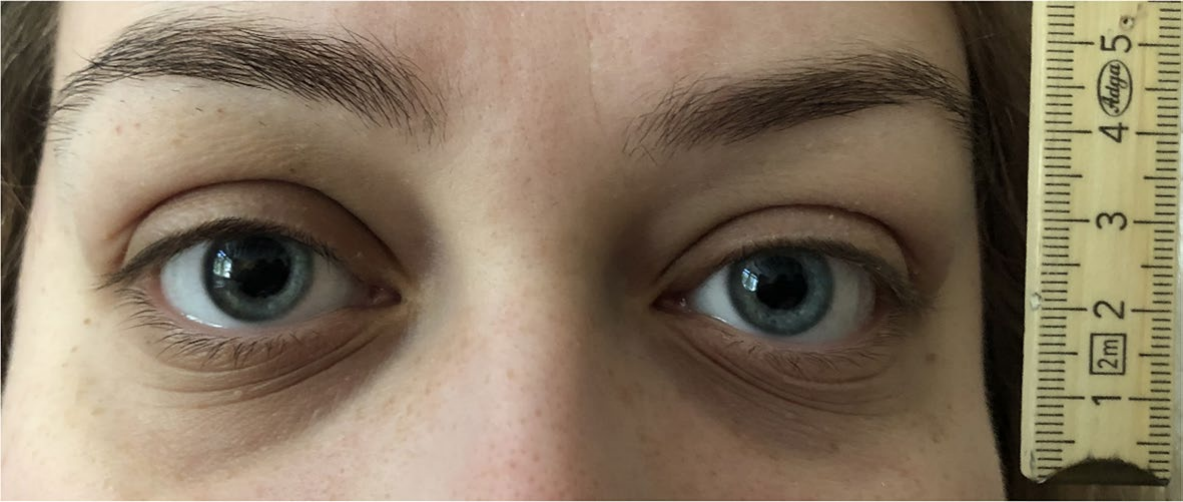
Dilated pupils on admission

A previously healthy 23-year-old woman presented with bilateral mydriasis (pupil diameter: left 6 mm, right 8 mm, both unresponsive to direct and indirect light stimuli) (Fig. 1). The mydriasis on the right side had occurred 6 weeks prior to admission and had persisted since. The mydriasis on the left side has been noticed by the patient on the day of admission. Further neurologic examination, including ocular motility, remained without any abnormalities.

MRI scans of the brain 4 weeks prior to admission and on day of admission conducted with gadolinium contrast were unremarkable.

MRI scans of the cervical spine and chest 15 days prior to admission showed enlarged lymph nodes in the mediastinum and supraclavicular on both sides.

The result of histopathologic examination of a lymph node biopsy specimen showed classical Hodgkin’s lymphoma.

**Fig. 2:**
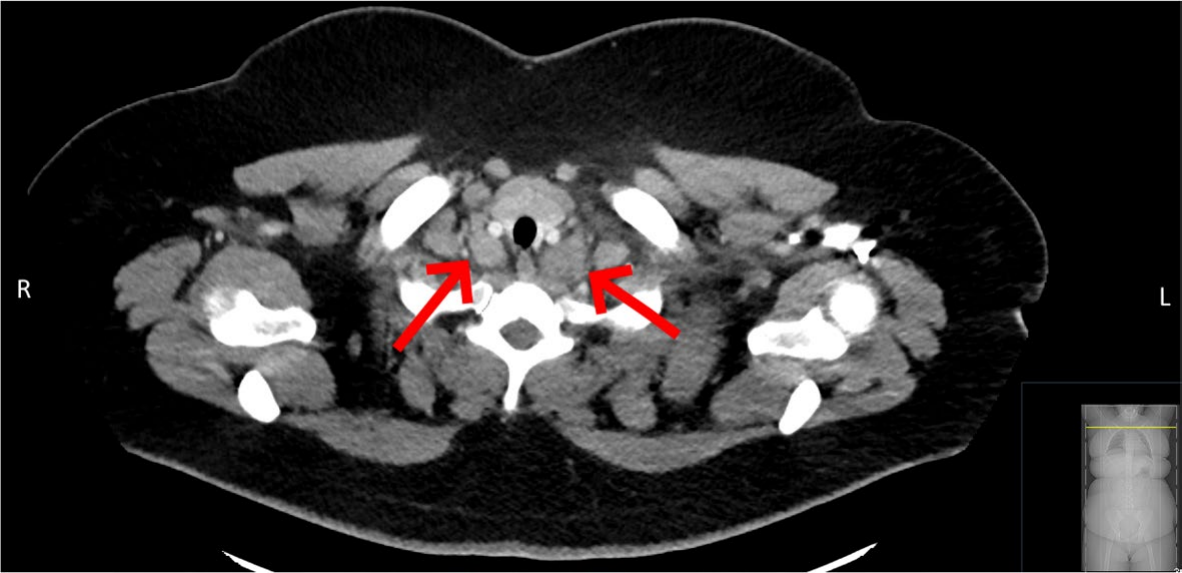
Enlarged lymph nodes in close proximity to the the carotid artery on both sides

^18^F-FDG PET/CT performed for staging revealed further pathological lymph nodes on the left axillary, the right pelvic area and retroperitoneal. Mediastinal and cervical lymph nodes were found significantly enlarged compressing the carotid artery on both sides (Fig. 2).

Cerebrospinal fluid (CSF) examination demonstrated a lymphocytic pleocytosis (10 cells/μl) with normal protein and glucose levels. Polymerase chain reaction (PCR) for herpes simplex virus (HSV) -1 and − 2 was positive with low genome concentration (< 500 genome copies/ml). Flow cytometric analysis of CSF leukocytes showed a normal cell distribution and no signs of meningeosis lymphomatosa.

Therefore, the patient was treated with 18 day acyclovir therapy and a chemotherapy with escalated BEACOPP (bleomycin, etoposide, doxorubicin, cyclophosphamide, vincristine, procarbazine, prednisone). After approximately four months and four cycles of escalated BEACOPP a new ^18^F-FDG PET/CT for follow-up assessment and restaging showed a complete remission*.* There was a slight improvement of the bilateral mydriasis.

## Discussion and conclusions

We describe the case of a patient presenting with bilateral mydriasis and first diagnosis of Hodgkin’s lymphoma.

Pathophysiologically, we consider the enlarged lymph nodes as irritating the pericarotid sympathetic nerves, causing oculosympathetic spasm and ultimately resulting in autonomic dysfunction clinically manifested by pupillary dilation.

The pupillary dilator muscle is innervated by the sympathetic nervous system [6]. The first neuron of this pathway is located in the hypothalamus. Exiting axons descend uncrossed through the brainstem tegmentum into the spinal cord to the level of C8–T1, where the second-order fibers begin and ascend in the sympathetic chain over the pulmonary apex to the superior cervical ganglion.

Postganglionic sympathetic fibres ascend from the superior cervical ganglion, along the walls of the internal carotid artery and innervate the ciliary ganglion activating the pupillary dilator muscle.

Pathologies of the brain stem as a potential differential diagnosis were clinically unlikely since all other brain stem reflexes, like the corneal reflex, remained intact and were eventually excluded by two unremarkable MRI scans of the brain.

We assume the detection of HSV-DNA in the CSF with low genome concentration to be a reactivation, possibly due to altered immune system in Hodgkin’s lymphoma disease, and in no causal link to the mydriasis.

The mild improvement of the mydriasis after the start of chemotherapy supports our hypothesis. However, as in most observational case studies a causality cannot be proved with absolute certainty.

Neurologic complications from Hodgkin’s lymphoma are overall rare. Direct neurologic dysfunction can be caused by metastases of the central nervous system and depending on the site of metastasis result in cranial nerve palsies, headaches and gait disturbance. Indirect affection of the central or peripheral nervous system by paraneoplastic syndromes can cause cerebellar degeneration and clinically present with dysarthria, nystagmus and ataxia [7]*.*

The case presented herein shows a bilateral mydriasis as the primary clinical manifestation of Hodgkin’s lymphoma.

To the best of our knowledge, this is the first reported case of mydriasis in association with Hodgkin’s lymphoma. Other reports described Horner syndrome in a patient with Hodgkin’s lymphoma with enlarged mediastinal lymph nodes [8] and oculosympathetic spasm with unilateral, intermittent mydriasis in a case of dissection of the internal carotid artery [5].

In conclusion, this case emphasizes the importance of detailed diagnostic, especially imaging of structures around the sympathetic pathway, in cases of autonomic dysfunction such as mydriasis.

## Data Availability

The data and images used in this case report are available from the corresponding author on reasonable request.

## Abbreviations

*18F-FDG PET*: Fluorine-18 fluorodeoxyglucose positron emission tomography
*BEACOPP*: Bleomycin, etoposide, doxorubicin, cyclophosphamide, vincristine, procarbazine, prednisone
*CSF*: Cerebrospinal fluid
*CT*: Computed tomography
*DNA*: Deoxyribonucleic acid
*HSV*: Herpes simplex virus
*MRI*: Magnetic Resonance Imaging
*PCR*: Polymerase chain reaction
